# From gene to therapy: a review of deciphering the role of *ABCD1* in combating X-Linked adrenoleukodystrophy

**DOI:** 10.1186/s12944-024-02361-0

**Published:** 2024-11-11

**Authors:** Xinxin Zuo, Zeyu Chen

**Affiliations:** 1 Department of Neurosciences, University of California San Diego, 9500 Gilman Drive, La Jolla, CA 92093 USA; 2 Department of Medicine, University of California San Diego, 9500 Gilman Drive, La Jolla, CA 92093 USA

**Keywords:** X-linked adrenoleukodystrophy (X-ALD), Very-long-chain fatty acids (VLCFAs), Genetic mutations, *ABCD1*, Neurological decline, Gene therapy

## Abstract

X-linked adrenoleukodystrophy (X-ALD) is a severe genetic disorder caused by *ABCD1* mutations, resulting in the buildup of very-long-chain fatty acids, leading to significant neurological decline and adrenal insufficiency. Despite advancements in understanding the mechanisms of X-ALD, its pathophysiology remains incompletely understood, complicating the development of effective treatments. This review provides a comprehensive overview of X-ALD, with a focus on the genetic and biochemical roles of *ABCD1* and the impacts of its mutations. Current therapeutic approaches are evaluated, discussing their limitations, and emphasizing the need to fully elucidate the pathogenesis of X-ALD. Additionally, this review highlights the importance of international collaboration to enhance systematic data collection and advance biomarker discovery, ultimately improving patient outcomes with X-ALD.

## Introduction

X-linked adrenoleukodystrophy (X-ALD) is a genetically mediated disorder that results in progressive deterioration of the central and peripheral nervous systems and the adrenal cortex. It predominantly affects male patients and is estimated to be present in approximately 1 in 17,000 newborns [1]. X-ALD manifests in three phenotypes, which vary according to the age of onset and the severity of symptoms: cerebral ALD (CALD), which manifests as childhood cerebral (CCALD) in those aged 4–10, adolescent cerebral (adolescent CALD) in those aged 10–20, and adult cerebral (adult CALD) in those aged over 20; Adrenomyeloneuropathy (AMN), which is characterized by spinal cord demyelination and axonal degeneration; and Addison-like X-ALD, which presents as adrenocortical insufficiency [2]. CCALD and AMN are the most prevalent forms of X-ALD. CCALD typically presents in boys aged 2.5–10 and is marked by inflammatory processes in cerebral white matter, leading to demyelination. This progression results in spastic quadriparesis, visual impairment, dysphagia, and eventually a vegetative state [3]. CCALD is the most severe form of cerebral ALD, as without intervention, it results in progressive lesions in white matter, leading to significant disability and then death, while AMN involves the chronic progression of axonopathy and primarily manifests as spinal cord dysfunction and peripheral neuropathy. AMN symptoms usually begin to appear in men in their 30s and 40s, whereas AMN clinical signs may appear in women after the age of 60 [4]. AMN is categorized into “AMN pure” and “AMN cerebral” types [5]. In AMN pure, pathology is confined to the spinal cord, manifesting as ambulatory disturbances and urinary dysfunction. AMN cerebral exhibits the spinal cord involvements seen in AMN pure and additional cerebral inflammatory symptoms. The mechanisms underlying the transition from AMN pure to AMN cerebral are not clearly defined.

X-ALD results from mutations in *ABCD1*, which encodes the ATP-binding cassette (ABC) subfamily D member 1 (ABCD1), a 745-amino-acid peroxisomal transmembrane protein [6]. ABCD1 is crucial for transporting very-long-chain fatty acids (VLCFAs) into peroxisomes, where they undergo β-oxidation [6]. In X-ALD, mutations in *ABCD1* impair this process, resulting in the accumulation of VLCFAs in the blood and in tissues, such as adrenal gland, testicular, and nervous system tissues. The most common diagnostic methods involve measuring the total concentration of the VLCFA C26:0 or the ratios of C26:0 to C22:0 and C24:0 to C22:0, as concentrations of C22:0 typically remain stable in patients with X-ALD [7]. However, despite extensive research, the exact correlation between specific gene mutations and symptoms remains undetermined, and VLCFA concentrations are not reliably predictive of disease-specific symptoms such as adrenal insufficiency or neurological decline. Current treatments focus on genetic repair or mitigating secondary effects, such as oxidative stress.

This review will examine the genetic and biochemical functions of ABCD1, with a focus on analyzing *ABCD1* mutations using data from the X-ALD mutation database to explore their distribution, density, and functional significance. Current therapeutic strategies for X-ALD will be evaluated, noting their limitations and the need for further research into the disease’s pathogenesis. Finally, this review emphasizes the value of global collaboration in advancing gene therapy research and improving patient outcomes in X-ALD.

## Molecular pathophysiology of X-ALD

The ABC transporter family comprises membrane proteins that are found across all organisms, from bacteria to humans. These transporters generally have a tetrameric structure that consists of two nucleotide-binding domains (NBDs), which hydrolyze ATP, and two transmembrane domains (TMDs), which transport a variety of substances across cellular membranes. These substances include simple molecules (such as amino acids, nucleosides, fatty acids, and sugars) and more complex molecules (such as proteins, polysaccharides, oligonucleotides, and lipids) [8]. However, ABCD1 has only one NBD and one TMD and thus must undergo dimerization (i.e., either homo- or heterodimerization) to form a fully functional transporter on the peroxisomal membrane [9]. VLCFAs are exclusively metabolized through β-oxidation within peroxisomes, making these organelles critical, particularly in the brain, where the lipid composition of myelin is crucial for its proper function and stability [10]. β-oxidation degrades fatty acids via the following four steps: a first dehydrogenation step (in which a hydrogen atom is removed), a hydration step (in which a water molecule is added), a second dehydrogenation step, and a thiolytic cleavage step. The last step cleaves the fatty acid chain, releasing a shortened fatty acid and acetyl-CoA [11]. In X-ALD, impaired ABCD1 functioning leads to the accumulation of VLCFAs, which markedly affect the adrenal glands, testes, brain, and spinal cord. This accumulation, particularly of C26:0 and C26:1, disrupts cellular and myelin membranes, leading to oxidative stress and neuroinflammation, and subsequently to neurodegeneration and cell death. These disruptions particularly affect oligodendrocytes, which are essential for myelination, thereby exacerbating the symptoms of X-ALD [12].

In addition to ABCD1, the ABCD family has three other members: ABCD2, ABCD3, and ABCD4. ABCD2 has a high degree of sequence homology with ABCD1 and plays a role in transporting both saturated and unsaturated VLCFA-CoAs into peroxisomes [13]. While ABCD1 predominantly targets saturated VLCFAs, such as C24:0-CoA and C26:0-CoA, ABCD2 has a greater affinity for polyunsaturated VLCFAs like C22:6-CoA and C24:6-CoA [14]​. ABCD1 and ABCD2 can form homodimers and heterodimers in peroxisomal membranes, with both dimeric forms being functional [9]. The specific function of ABCD2 in vivo remains uncertain, as it has a low level of natural expression in human cells and its dysfunction has not been linked to any diseases. ABCD3 is most abundant on peroxisomal membranes, especially in hepatocytes, and typically exists as a homodimer. Experiments in ABCD1-deficient U87 cells involving the silencing of ABCD3 demonstrated that ABCD3 has minimal involvement in the β-oxidation of C24:0, which occurs independently of ABCD1 [15]. ABCD3 may play a role in transporting hydrophilic substrates, such as bile acid intermediates and long-chain fatty acids (LCFAs), rather than VLCFAs. ABCD4 is localized to lysosomes and is believed to be essential for the transfer of vitamin B12 from lysosomes to the cytosol [16].

## Mutations in *ABCD1*

In 1993, Aubourg and co-workers used positional cloning to identify that mutations in *ABCD1* are the cause of X-ALD [17]. *ABCD1* is located on the Xq28 band of the X chromosome, is 19.9 kb long, and contains 10 exons. Mutations in *ABCD1* have been universally identified in patients diagnosed with X-ALD, with a comprehensive catalog available in the X-ALD database (https://adrenoleukodystrophy.info/; last consulted on August 1, 2024). Thus far, more than 950 disease-causing mutations have been reported, consisting of missense mutations (41.1%), frameshift mutations (30.6%), nonsense mutations (13.7%), amino acid insertion or deletion mutations (5.8%), splice-site mutations (5.0%), single or multiple exon deletion mutations (2.6%), and more than 120 variants of uncertain significance (VUSs). As an ABC protein, ABCD1 contains four functional domains: a peroxisomal biogenesis factor 19 (PEX19)-binding domain, which is involved in protein–protein interactions; a TMD with six transmembrane helices; an NBD featuring Walker A and B motifs and the ABC signature sequence; and a dimerization domain. PEX19, a chaperone protein, engages with a wide array of peroxisomal membrane proteins and plays a crucial role in the peroxisomal targeting of ABCD1 by interacting with its PEX19-binding domain [18]. Most missense mutations affect ABCD1 stability and result in its degradation [19]. Moreover, while the majority of X-ALD patients inherit a defective *ABCD1* allele from a parent, as many as 19% of cases of X-ALD are attributable to de novo mutations [20].

**Fig. 1:**
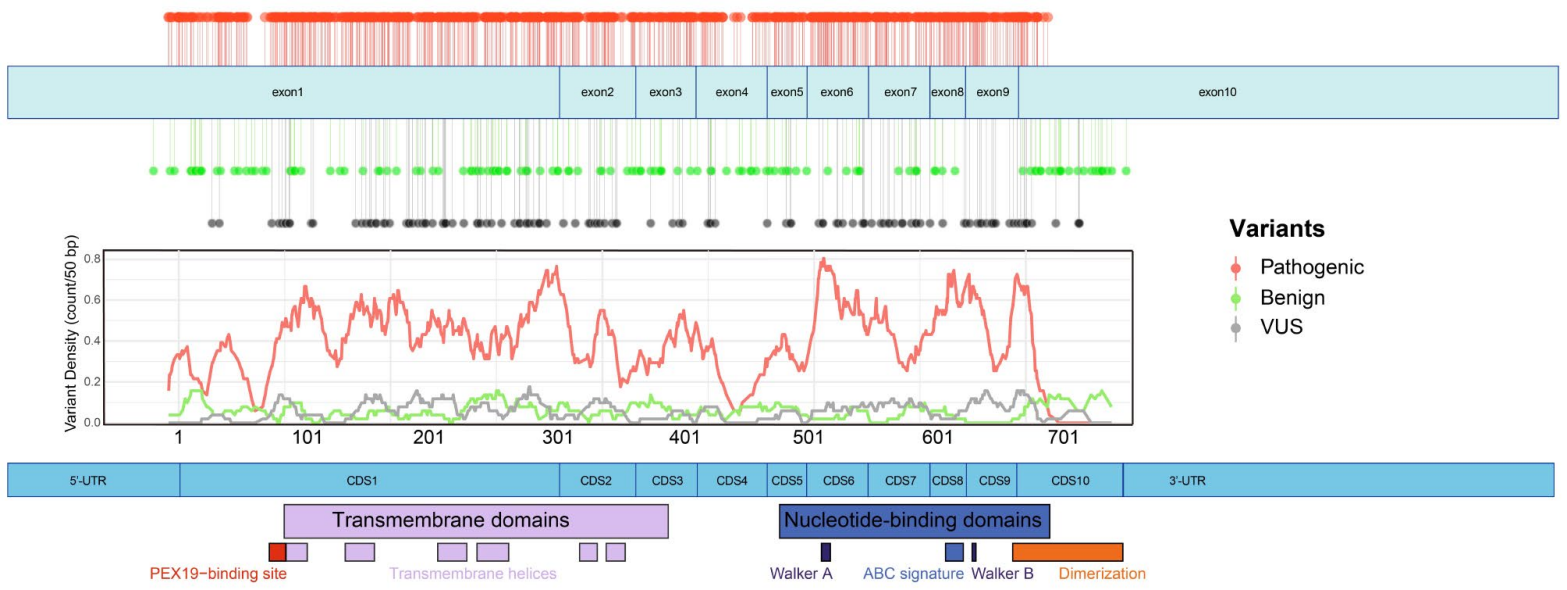
Distribution of Mutations of *ABCD1* and Functional Domains of ABCD1. The top panel depicts the distribution of mutations in *ABCD1* (excluding introns), categorized as pathogenic or likely pathogenic mutations (colored red), benign or likely benign mutations (colored green), or variants of uncertain significance (VUSs) (colored gray). The middle panel contains a variant density plot based on a 50-base-pair sliding window across *ABCD1*, with the *x*-axis representing the positions of amino acids. The bottom panel highlights the protein domains encoded by *ABCD1*. CDS = Coding Sequence, UTR = Untranslated Region. Mutation data was obtained from https://adrenoleukodystrophy.info/

Integrating data from the ABCD1 database (https://adrenoleukodystrophy.info/), we analyzed the mutation density, types, and functional distribution within *ABCD1* (Fig. 1). Analysis of missense mutations has revealed that they are distributed non-randomly across *ABCD1*. Peak densities of missense mutations have been found in the TMD in exons 1 and 2, which encode six transmembrane helices, and in exons 6–9, which encode the ATP-binding domain. The peak density of pathogenic mutations has been found in the NBD in exon 6 (in the Walker A domain), with high densities also found in exon 8 (in the ABC signature sequence) and in exon 9 (in the Walker B domain). The NBD encodes structures responsible for ATP binding and hydrolysis, and these processes provide energy for substrate transport, which is the core function of ABCD1. In addition, a high density of mutations has been found in the region encoding the linker between the fourth and fifth transmembrane helices within the TMD. This linker is located on the cytoplasmic side of ABCD1 and in close physical proximity to the NBD and thus is hypothesized to be critical for the stability and function of ABCD1. Moreover, a peak density of pathogenic mutations has been found in the region encoding amino acids (aas) 656–668 of the N-terminal region of the dimerization domain. This region is immediately downstream of the region encoding the NBD. Thus, the pathogenicity of mutations in this region indicates that it encodes the core of the dimerization domain. In contrast, the density of mutations is significantly lower in regions encoding other parts of the dimerization domain.

Interestingly, although the coding sequences of *ABCD1* extend to position aa 745, no disease-causing mutations have been identified beyond aa 684. Moreover, the sequence beyond aa 684 in ABCD1 shows a greater prevalence of benign mutations than other regions, indicating that this segment may not play a direct role in substrate transport. This possibility is supported by the fact that a truncated version of ABCD1, consisting of aas 1–693, restored β-oxidation of VLCFAs in fibroblasts from X-ALD patients [21]. In addition to the region of *ABCD1* encoding the C-terminus of ABCD1, two other regions of *ABCD1* exhibit low densities of pathogenic mutations. One is the region encoding the upstream area of the PEX19-binding site (aas 57–69) at the N-terminus. This region also has a considerable number of benign mutations, indicating that it encodes parts of ABCD1 that are not critical for its functioning. For example, a 26-base-pair deletion that results in removal of the ATG translation initiation codon of *ABCD1* leads to internal initiation of translation to afford a truncated ABCD1 (i.e., lacking the first 65 aas of its N-terminus) [22]. This mutant ABCD1 is correctly trafficked to peroxisomes but results in β-oxidation of VLCFAs being reduced to 20% of its normal level. In addition, no instances of translation initiation mutations in ABCD1 have been associated with CALD; instead, such mutations have exclusively resulted in AMN. Another region with a low density of pathogenic mutations is in exon 4. This region encodes the linker between the TMD and NBD and thus probably has a considerable degree of structural flexibility. In summary, it appears that the density of benign mutations in a region of *ABCD1* can indicate the functional importance of the area of ABCD1 the region encodes. Specifically, the aforementioned three regions have low densities of pathogenic mutations but rather high densities of benign mutations. This observation suggests that compared with mutations in other regions, mutations in these three regions are better tolerated, due to these regions encoding areas of ABCD1 that are not critical to its functioning.

X-ALD is a complex disease, as the same *ABCD1* mutation can lead to a variety of phenotypes, even among individuals with similar genetic backgrounds and environmental conditions. This variability is evident in clinical observations, such as in the case of a set of monozygotic twins: one twin remained symptom-free, while the other developed symptoms such as gait ataxia, visual impairments, and cerebral demyelination [23]. In addition, it was recently reported that two Japanese siblings shared a novel *ABCD1* missense mutation (c.1887T > G) but had different clinical outcomes. The first sibling experienced severe symptoms and died at age 25, whereas the second sibling showed milder symptoms, which began at age 22 [24]. The aforementioned two cases underscore the unpredictable nature of X-ALD and that there is not a direct correlation between specific mutations and disease severity.

## Current therapeutic strategies

### Hematopoietic stem-cell transplantation (HSCT)

HSCT was first explored for treating X-ALD in the 1980s and has involved significant challenges and setbacks. For example, one young patient with X-ALD underwent successful engraftment but succumbed to disease progression 141 days later [25]. In 1990, Aubourg et al. described an application of HSCT for an 8-year-old male patient diagnosed with X-ALD following an adrenal crisis [26]. The patient demonstrated alterations in the white matter of the internal capsule and exhibited deficits in attention, verbal skills, and memory. His fraternal twin brother served as the stem cell donor. Post-transplantation, the patient showed only slight neurological progression, accompanied by a reduction in plasma VLCFA concentrations and enhanced C24:0 degradation. These outcomes indicated that the transplanted cells from the donor had breached the blood–brain barrier and contributed to the stabilization of demyelination. This case provided early evidence of the significant benefits of timely HSCT in changing the trajectory of X-ALD and improving patient prognoses.

The Loes score, developed by Loes and colleagues in 1994, is the gold standard for assessing the severity of CALD using MRI (magnetic resonance imaging). This scoring system, with a maximum of 34 points, is based on the location and extent of brain involvement, as well as the presence of focal or global atrophy [27]. MRI changes often precede clinical signs, enabling early diagnosis and timely therapeutic intervention. A score below 4 indicates a very early stage, 4 to 8 represents early disease, 9 to 13 suggests late-stage, and scores above 13 indicate advanced disease. HSCT is the established treatment for CALD in patients with a Loes score of less than 9 [28], offering potential to slow neuroinflammatory demyelination when performed early, although the timing of stabilization may vary depending on individual patient response [29]. Prompt recognition of cerebral symptoms after newborn screening may mitigate the limited efficacy of HSCT in boys with CALD. However, it is challenging to achieve timely implementation of HCST in adults because compared with CALD in children, CALD in adults exhibits subtler demyelination and symptom onset, and may be subject to less frequent monitoring by MRI. Given the current lack of phenotype-specific biomarkers, such biomarkers must be developed to refine the identification of suitable candidates for HSCT. Furthermore, mortality rates in patients receiving HSCT vary: from 5 to 20% in children to 20–40% in adults, when using donors that are reasonably good human leucocyte antigen matches with the patient, employing complete myeloablation, and therapy with cyclophosphamide and busulfan, depending on how advanced the disease is [30]. Moreover, HSCT is associated with the risk of graft-versus-host disease and prolonged immune deficiency.

### Advances in gene therapy for X-ALD

#### Lentiviral (LV)-based hematopoietic stem-cell gene therapy

To reduce the morbidity associated with allogeneic HSCT and address the lack of matched donors, treatment with LV-based hematopoietic stem-cell gene therapy has been explored. Cartier et al. performed the first tests of this form of gene therapy in human patients with cerebral disease [31]. They treated two boys with CALD using LV-mediated gene transfer into mobilized peripheral blood stem cells (CD34^+^). These cells were harvested, corrected ex vivo with a vector containing wild-type ABCD1, and reinfused post-myeloablative treatment. Over a 24–30 month follow-up, polyclonal reconstitution was achieved, with 9–14% of various hematopoietic cells expressing ABCD1, demonstrating effective stem cell transduction. From 14 to 16 months post-infusion, cerebral demyelination ceased, yielding clinical outcomes comparable to those achieved with allogeneic HSCT. Following this investigation, two additional reports were published, covering a total of four patients [32]. The most recent report comprised a follow-up of more than 8.8 years and found that one patient had exhibited a near-normal cognitive status for over 8 years, while the other three patients had exhibited significant declines in cognitive status over this period [33]. MRI scans showed that initial brain lesions had resolved within a year, yet genetic analyses revealed that therapeutic protein expression had decreased, stabilizing at approximately 5–10%. These results demonstrate that while this hematopoietic stem-cell gene therapy showed safe and stable integration, cognitive deterioration persisted in most patients. Consequently, there is a need for the development of earlier-stage and more potent therapeutic interventions.

In another example, modified LV vectors were developed and used to reduce the risk of mutagenesis. The Lenti-D construct is a replication-defective LV vector, similar to that used in the trial discussed above, and utilizes a modified MLV long terminal repeat (MNDU3) promoter to express *ABCD1*. In 2017, 17 boys with CALD received Lenti-D gene therapy, which was shown to be safe and effective over a median follow-up of 29.4 months [34]. All patients demonstrated engraftment of gene-marked cells without adverse integration patterns or clonal expansion. Moreover, all patients maintained detectable concentrations of ABCD1, with no instances of treatment-related mortality or graft-versus-host disease. Ultimately, 88% of the patients remained alive without significant functional disabilities and showed only minimal clinical symptoms.

#### CRISPR/Cas9-mediated in vivo gene editing

In addition to work on ex vivo LV gene correction, efforts are also focused on developing in vivo gene therapy using adeno-associated virus serotype 9 (AAV9) vectors. Gong et al. showed that in mouse models, AAV9 effectively delivers human *ABCD1* to the central nervous system (CNS) and adrenal glands and significantly reduces concentrations of VLCFAs [35]. This gene therapy targets various cells in the CNS, including neurons, astrocytes, and microvascular endothelial cells. In a subsequent study, the optimized method involved the use of an osmotic pump to deliver the vector slowly and directly into the lumbar cerebrospinal fluid. This delivery method enhanced the distribution and expression of *ABCD1* across the spinal cord while minimizing systemic leakage and potential peripheral toxicity [36]. Similarly, other researchers used direct brain injections of an LV *ABCD1* vector and found that these led to robust *ABCD1* expression around injection sites without causing damage to neurons or immune response [37]. Specifically, these researchers created an X-ALD knockout (KO) mouse model using a CRISPR–Cas9 system and observed behavioral and motor improvements after injections into targeted brain areas. These results established an early-onset X-ALD model and demonstrated that it was possible to achieve significant neurological recovery post-injection that was free from immunopathological toxicity.

Intravenous or intracerebroventricular administration of AAV9-delivered exogenous *ABCD1* can achieve continuous expression of ABCD1, but in concentrations that are inconsistent with those of endogenous ABCD1, which is suboptimal for treating X-ALD. Therefore, targeted correction of endogenous ABCD1 is considered a more precise approach. Recently, homology-independent targeted integration (HITI) was utilized to treat X-ALD [38]. This strategy, combined with base editing, corrected ABCD1 in fibroblasts from a male patient with a point mutation (c.796G > A, p.Gly266Arg). Additionally, in an X-ALD mouse model, HITI-AAV vectors enabled precise integration of human *ABCD1* near the 5′-UTR of *Abcd1*. Treated mice showed significant *ABCD1* mRNA expression and reduced concentrations of the diagnostic markers C24:0-LysoPC (Lysophosphatidylcholine) and C26:0-LysoPC. In a 2024 study, researchers established a humanized mouse model of X-ALD by integrating human cDNA with the p.G512S mutation (c.1534G > A) into the murine *Abcd1* locus, resulting in increased VLCFA concentrations [39]. In addition, by using base and prime editing techniques, they achieved a 7.4% correction rate in patient-derived fibroblasts. Administering the base editor via AAV corrected the mutation in various tissues, i.e., by 5.5% in the brain, 5.1% in the spinal cord, and 2% in the adrenal gland, resulting in a marked decrease in the plasma C26:0-to-C22:0 ratio. This model and its effective correction represent a major advance in X-ALD treatment.

### Hormone replacement therapy

Primary adrenal insufficiency occurs in up to 86% of male patients with X-ALD, while female carriers are less commonly affected [40]. It presents with symptoms such as fatigue, anorexia, nausea, and increased skin pigmentation, and may also cause hair loss. It is treated with chronic glucocorticoid replacement therapy, with hydrocortisone typically used in children to minimize growth impacts, and prednisolone or prednisone used in adults. Doses are adjusted based on symptoms and plasma concentrations of adrenocorticotropic hormone [41]. Moreover, during periods of increased physiological stress, doses are increased, and during acute crises, immediate administration of hydrocortisone, intravenous fluids, and dextrose is crucial. Less common symptoms of primary adrenal insufficiency are mineralocorticoid deficiencies, which are manifested as salt-craving behaviors. If left untreated, mineralocorticoid deficiencies result in biochemical changes, namely hyponatremia, hyperkalemia, mild metabolic acidosis, and increased plasma renin activity [42].

### Dietary therapy

Dietary interventions such as low-fat diets can decrease plasma concentrations of VLCFAs in patients with X-ALD but do not change the overall disease trajectory [43]. Patients, particularly male patients with adrenal insufficiency, are advised to manage their intake of VLCFA-rich foods, such as vegetable oils, seeds, and fatty fish [44]. They may also need to avoid licorice and grapefruit juice, which affect cortisone metabolism, and if they have mineralocorticoid deficiency, they should aim to maintain an adequate salt intake [41]. Lorenzo’s oil, a 4:1 mixture of oleic and erucic acid triglycerides, was initially explored as a potential treatment for X-ALD. While it has been shown to normalize VLCFA concentrations in the plasma, it has not shown neurological or endocrine improvements in clinical settings [45]. Some non-placebo-controlled trials have suggested that Lorenzo’s oil protects against the development of CALD in asymptomatic patients. However, more comprehensive evaluations, including double-blind placebo-controlled trials, demonstrated that Lorenzo’s oil not significantly halt or slow the progression of X-ALD symptoms in patients with AMN or CALD [46].

### Antioxidant therapy

Oxidative damage is an important early contributor to the pathology of X-ALD and is particularly linked to axonal degeneration [47]. Research on antioxidant treatment in models of X-ALD that mimic the late-onset neurological symptoms and locomotor disabilities typical of the AMN phenotype have yielded promising results. In particular, treatments consisting of a combination of antioxidants, such as *N*-acetyl-cysteine, α-tocopherol, and α-lipoic acid, have proven effective. These antioxidants helped to mitigate oxidative damage and enhanced locomotor abilities in mice deficient in both *Abcd1* and *Abcd2* [48]. A phase II pilot study involving 13 patients with myelopathy explored the biological effects of high-dose antioxidant therapy over 3 months [49]. This regimen led to a marked reduction in the concentrations of pro-inflammatory markers in both plasma and peripheral blood mononuclear cells, suggesting that oxidative damage and inflammation had been alleviated. Interestingly, the patients also appeared to demonstrate improved performance in the 6-Minute Walk Test. Other researchers found that dimethyl fumarate (DMF), a drug known for its antioxidant properties, was effective in preventing oxidative damage in a mouse model of AMN [50]. Specifically, DMF treatment enhanced the activity of the nuclear factor erythroid 2-related factor 2 (Nrf2) pathway and the antioxidant system and reduced inflammation in the spinal cord. Moreover, DMF treatment of an *Abcd1/2* double KO mouse model of X-ALD reversed signs of astrocytosis, microgliosis, and axonal and myelin degeneration, and enhanced locomotor function. Recently, it was found that high-dose biotin significantly improved redox balance, mitochondrial function, and lipid metabolism in mouse models of X-ALD [51]. Specifically, high-dose biotin restored mitochondrial biogenesis and ATP concentrations, corrected lipid dysregulation by normalizing mTORC1/SREBP-1c signaling, and reduced lipid-droplet accumulation. These biochemical improvements were accompanied by enhanced motor functions and axonal integrity, suggesting that high-dose biotin is a promising therapy for X-ALD.

## Discussion

### Challenges and limitations of current X-ALD therapies

While HSCT remains a standard treatment for CCALD, uncertainties persist regarding its long-term effectiveness and the risks of complications like graft-versus-host disease. More clinical trials are needed to compare the outcomes of gene therapy and traditional HSCT, particularly for adult patients with AMN. A gene therapy developed by bluebird bio, Skysona™ (also known as Lenti-D™), has been approved in the European Union and the United States (U.S.) and targets boys aged 4 to 17 with early and active CALD [52]. Clinical trials of Skysona™, including trials ALD-102 and ALD-104, have demonstrated that most patients maintain major functional disability-free survival and stable neurological function scores for 24 months [52]. While Skysona™ has shown promise in treating X-ALD, its potential to cause hematologic malignancies, such as myelodysplastic syndrome, warrants close monitoring. Alternatively, AAV therapies offer a safer, non-integrative approach with reduced immunogenicity, making them ideal for non-dividing cells. However, most of their benefits are temporary, highlighting the need for further development to achieve long-term efficacy. Thus, the advantages and limitations of various treatments require ongoing assessment to enhance safety and effectiveness.

Most HSCT-based research and clinical trials focused on CALD, especially in children, where progression is more severe and rapid. However, there fewer HSCT-based studies and clinical trials focused on AMN, primarily because it generally presents later in life and has a slower progression [53]. Additionally, HSCT is less effective in AMN than in CALD, likely due to there being less inflammatory activity in AMN than in CALD [54]. In 2024, Siwek et al. reported that intrathecal delivery of mesenchymal stem cells (MSCs) derived from Wharton’s jelly significantly improved motor function in patients with AMN [55]. Key findings included marked increases in muscle strength in the lower limbs and increased walking speeds, indicating that this may be a viable treatment for managing symptoms of AMN. Additionally, the treatment was generally safe and well tolerated, with only minor, transient side effects observed, supporting the potential of MSCs as a therapeutic option for AMN.

The mechanism by which HSCT stabilizes CALD is not fully understood. It is alternatively hypothesized that chemotherapy and immunosuppression integral to HSCT independently contribute to disease stabilization. However, when patients fail to engraft donor-derived cells after transplantation, they typically exhibit a return of neuroinflammation and further deterioration of white matter [56]. This observation underscores that ABCD1-producing cells are necessary for stabilizing CALD. Further investigation is required to determine if the advantages of these cells are due to their migrating to the brain, where they decrease VLCFA concentrations, reduce oxidative stress, and alleviate inflammation, or to other mechanisms. Finally, hematopoietic stem-cell gene therapy for CALD was recently reported to generate significant and sustained improvements in white matter permeability and microvascular flow [57]. Thus, this therapy effectively slowed the progression of CALD lesions, suggesting that modified cells contribute to the sustained reconstruction of brain microvascular function. It also suggests that improved microvascular flow resulted from the normalization of interactions between corrected endothelial cells and leukocytes, which may represent the mechanism of action of this therapy.

### Unraveling the complex pathogenesis of X-ALD

No definitive correlation between genotype and phenotype has been established in patients with X-ALD. Clinical phenotypes can vary even among monozygotic twins, and significant deletions or frameshift mutations can sometimes lead to mild phenotypes [20]. Furthermore, patients with X-ALD often progress through multiple phenotypes, complicating characterization of the disease. Although X-ALD is a monogenic disease, its severity and penetrance may be influenced by other genetic factors and environmental triggers. Research has investigated modifier genes that encode proteins involved in metabolic or immune pathways to determine how these genes impact X-ALD [2, 58]. *ABCD2* and the stearoyl-CoA desaturase 1 (*SCD1*) gene encode proteins involved in peroxisomal VLCFA metabolism; the cytochrome P450 family 4 subfamily F member 2 gene(*CYP4F2*) encodes a protein involved in ω-oxidation of VLCFAs; and the major histocompatibility complex class II DR beta 1 (*HLA-DRB1*), interleukin 6(*IL-6*), and tumor necrosis factor alpha (*TNF-α*) are genes encode proteins involved in the immune system. While some studies have explored potential associations between these genes and X-ALD phenotypes, their correlations have not been consistently demonstrated. Additionally, genes involved in methionine and folate metabolism, such as the methionine synthase and methylenetetrahydrofolate reductase genes, contribute to the complex interplay affecting the clinical manifestations of X-ALD [2]. Furthermore, the apolipoprotein E gene (*APOE*), which has three isoforms (*APOE2*, *APOE3*, and *APOE4*) encoded by three alleles at a single locus, plays a role in lipid transport. It was found that male patients with X-ALD carrying the *APOE4* allele showed more severe cerebral involvement (as indicated by MRI), poorer neurological function, and higher cerebrospinal fluid concentrations of matrix metalloproteinase-2 than those without the *APOE4* allele [59].

**Fig. 2:**
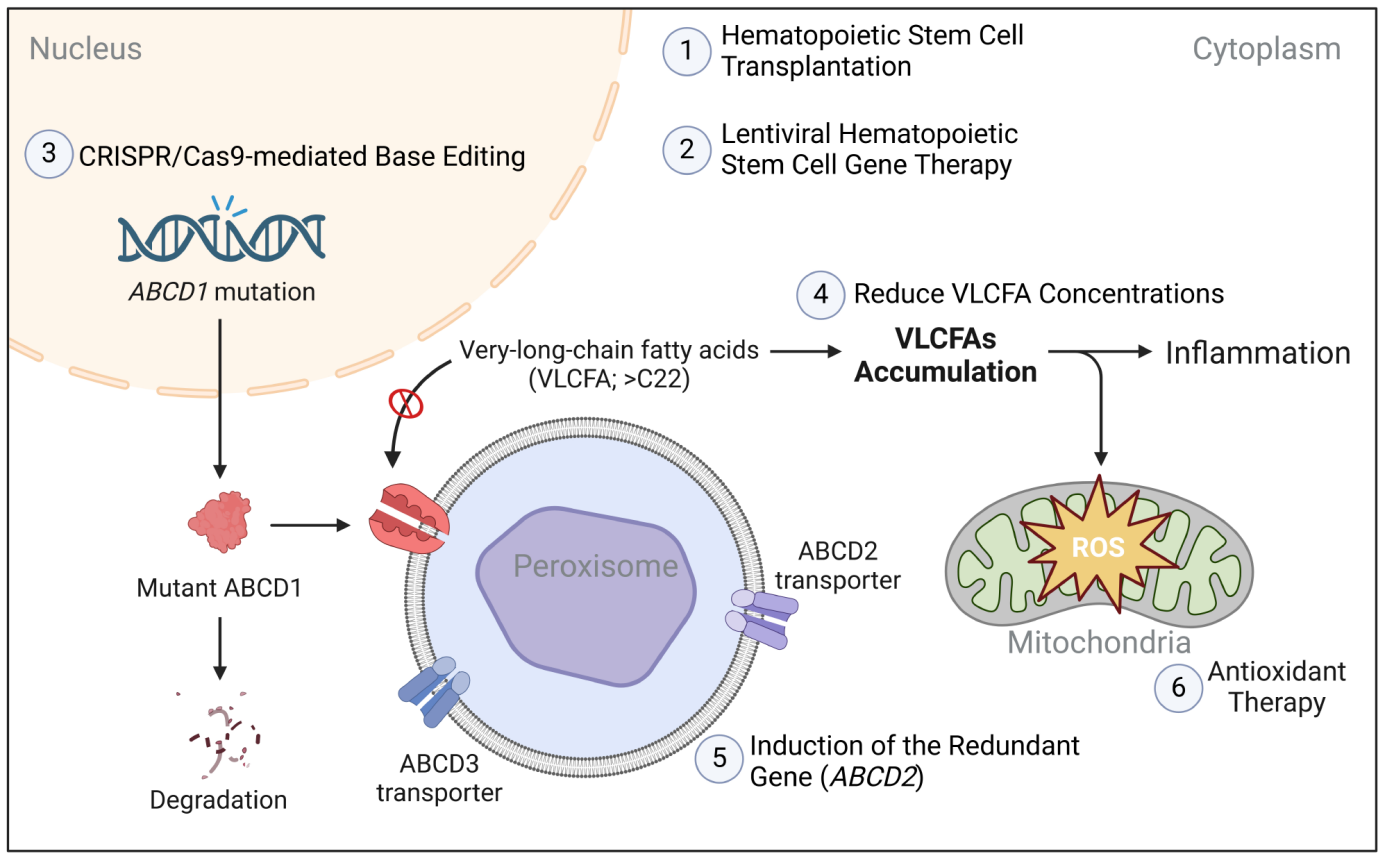
Therapeutic Targets in X-ALD Linked to *ABCD1* Mutations. The mutation disrupts normal ABCD1 functioning, leading to decreased β-oxidation of very-long-chain fatty acids (VLCFAs). This reduction leads to the accumulation of VLCFA-CoAs and thus an increase in concentrations of free VLCFAs. The accumulation of VLCFAs triggers oxidative stress and cellular damage. The figure indicates intervention points that must be addressed when developing treatments to target the underlying biochemical abnormalities in X-ALD. ROS = reactive oxygen species. The figure is created with BioRender.com

To advance developments in gene therapy, it is essential to understand the impact of ABCD1 overexpression in X-ALD. Over 75% of *ABCD1* mutations produce unstable, undetectable forms of ABCD1, while some stable missense *ABCD1* mutations disrupt function by reducing ATP-binding capacity or ATPase activity [60]. Moreover, the functionality of ABCD1 relies on its dimerization, and thus defective ABCD1 variants may exert a dominant-negative effect [61], i.e., reduce the functioning of normal ABCD1 molecules. Such an effect must be considered when devising gene therapy strategies, necessitating a decision between merely expressing a normal protein exogenously or performing in vivo gene editing. This decision should be tailored to the patient’s mutation to ensure precise treatment. Strategies have been developed that induce ABCD2, a peroxisomal transport protein, to compensate for dysfunctional ABCD1 [62]. However, these strategies may be influenced by the dominant-negative effect of mutant ABCD1, as this effect may decrease the functionality of potential ABCD1/ABCD2 heterodimers. In summary, the complex interplay of the abovementioned aspects indicates that further research is needed to clarify the mechanisms involved and optimize treatment strategies for X-ALD (Fig. 2).

Ongoing research into the pathogenesis of X-ALD faces challenges, including the limited availability of animal models that accurately replicate human disease progression. For example, the *Abcd1*-deficient mouse model primarily exhibits symptoms akin to AMN, such as late-onset axonopathy and locomotor impairments, but without the severe cerebral forms of X-ALD seen in humans [63]. More complex models, such as the *Abcd1*/*Abcd2 *double KO mouse model, show increased VLCFA accumulation and some neuropathic changes resembling human X-ALD, particularly in oligodendrocytes, but they fail to fully replicate human cerebral phenotypes [9]. Additionally, although these models show a late-onset spinal cord phenotype similar to that observed in women with X-ALD, they do not develop the neuroinflammation seen in human X-ALD. Recent advancements in zebrafish models have provided additional insights, as shown by Strachan et al., who demonstrated that the induction of human ABCD1 expression in oligodendrocytes in zebrafish abcd1 mutants reduced embryonic apoptosis, improved motor function, and partially rescued the observed deficits in oligodendrocyte patterning and myelination [64]. While this zebrafish model recapitulates some key features of human X-ALD, including VLCFA accumulation and motor impairments, it still does not completely capture the full spectrum of human X-ALD pathology. These limitations severely restrict their utility for testing potential therapies targeting the most critical aspects of X-ALD. Consequently, to advance our understanding of X-ALD and support the development of treatments, there is a pressing need for focused research and the development of new models that effectively mirror human X-ALD.

### Significance of newborn screening for X-ALD

X-ALD is a single-gene disorder linked to the X chromosome, thereby enabling precise calculation of the likelihood that affected parents will transmit a mutant *ABCD1* allele to their children. Sons of men with X-ALD will not inherit a defective *ABCD1* allele from their father. However, daughters of men with X-ALD are invariably carriers of a defective *ABCD1* allele. Both sons and daughters of women who are heterozygous carriers have a 50% chance of inheriting a defective *ABCD1* allele. While the majority of X-ALD patients inherit a mutant *ABCD1* allele from a parent, approximately 19% possess a defective *ABCD1* allele arising from a de novo mutation [20]. In 2006, Hubbard et al. introduced a method that uses liquid chromatography–tandem mass spectrometry (LC-MS/MS) to measure concentrations of C26:0–LysoPC in dried blood spots [65]. In 2013, New York became the first state in the U.S. to start screening newborns for X-ALD, following the implementation of Aidan’s Law [66]. In 2016, X-ALD was added to the Recommended Universal Screening Panel, and plans have since been made to expand X-ALD screening to all states [67]. As of 2024, approximately 44 states and Washington, D.C. test newborns for X-ALD.

Early diagnosis of X-ALD is crucial for timely treatment of cerebral involvement and/or adrenal insufficiency. However, predicting the phenotype remains a significant challenge due to the wide variability in disease manifestation, even among individuals with the same mutation. This unpredictability is compounded by the fact that genetic mutations in the *ABCD1* gene do not consistently correlate with clinical outcomes, and many variants of uncertain significance (VUSs) further complicate the interpretation [68]. Furthermore, newborn screening and subsequent cascade testing are expected to identify many female carriers—“patients in waiting”—who may remain asymptomatic for years but are at risk of developing symptoms later in life. This introduces challenges for long-term monitoring and care, along with potential concerns about uncertainty. At the same time, early identification allows families to make informed reproductive decisions, helping to reduce the risk of passing the condition to future generations.

### The imperative for international collaboration in X-ALD research

Determining the incidence or prevalence of X-ALD is complicated by several factors: the clinical diversity of the disease, the delayed emergence of symptoms, the involvement of small study cohorts, and differences in the characterization of affected families. Many patients are diagnosed late—often in stages where treatment options are limited—due to the substantial occurrence of de novo *ABCD1* mutations and the lack of a family history. The adaptation of newborn screening for X-ALD marks a significant advancement that ensures early diagnosis and ongoing monitoring of patients. However, this practice has not been widely adopted worldwide. Additionally, the lack of centralized record-keeping of heterozygous female patients and the misdiagnosis of mildly affected male patients in some countries can lead to an underestimation of the frequency of X-ALD. Therefore, the formation of worldwide collaborations for X-ALD research is imperative to enable systematic data collection and support detailed analytical evaluations [20]. Large consortia have been established, such as the European AMN Board in Europe and ALD Connect in the U.S., which actively involve patient organizations [49]. Such consortia play a critical role in assembling large-scale sample collections for genetic research and constructing solid frameworks for in-depth metabolic analyses [69].

Screening for X-ALD facilitates early intervention for adrenal insufficiency and cerebral demyelination, which can be lifesaving. However, challenges persist, due to a limited understanding of the fundamental progression of X-ALD and the absence of reliable prognostic biomarkers. Consequently, predicting disease progression is difficult, thereby complicating the development of effective monitoring strategies. The identification of biomarkers that allow early disease detection is a primary goal of research on other neurodegenerative disorders, such as Alzheimer’s disease and multiple sclerosis. Thus, examining research on these diseases can offer insights into potential biomarkers for X-ALD [70]. In conclusion, further research based on international collaborations between physicians, researchers, and patients is essential for enhancing the care of patients with X-ALD [68].

## Data Availability

The datasets analyzed during the current study are available in the public database: https://adrenoleukodystrophy.info/mutations-and-variants-in-abcd1.

## Abbreviations

X-ALD: X-linked adrenoleukodystrophy
VLCFAs: Very-long-chain fatty acids
ABCD1: ATP-binding cassette (ABC) subfamily D member 1
CALD: Cerebral ALD
AMN: Adrenomyeloneuropathy
VUS: Variant of uncertain significance
TMD: Transmembrane domain
NBD: Nucleotide-binding domain
HSCT: Hematopoietic stem-cell transplantation
MRI: Magnetic resonance imaging
CNS: Central nervous system
HITI: Homology-independent targeted integration
LysoPC: Lysophosphatidylcholine
